# A Quantitative PCR Protocol for Detection of *Oxyspirura petrowi* in Northern Bobwhites (*Colinus virginianus*)

**DOI:** 10.1371/journal.pone.0166309

**Published:** 2016-11-28

**Authors:** Whitney M. Kistler, Julie A. Parlos, Steven T. Peper, Nicholas R. Dunham, Ronald J. Kendall

**Affiliations:** The Wildlife Toxicology Laboratory, The Institute of Environmental and Human Health, Texas Tech University, Lubbock, Texas, United States of America; Defense Threat Reduction Agency, UNITED STATES

## Abstract

*Oxyspirura petrowi* is a parasitic nematode that infects wild birds. This parasite has a broad host range, but has recently been reported in high prevalences from native Galliformes species in the United States. In order to better understand the impact *O*. *petrowi* has on wild bird populations, we developed a quantitative PCR protocol to detect infections in wild northern bobwhites (*Colinus virginianus*). We used paired fecal and cloacal swab samples from wild caught and experimentally infected northern bobwhites and matching fecal float data from experimentally infected birds to validate our assay. Overall we detected more positive birds from fecal samples than the paired cloacal swabs and there was strong agreement between the qPCR results from fecal samples and from fecal flotation (84%; κ = 0.69 [0.53–0.84 95% CI]). We also detected *O*. *petrowi* DNA in ten replicates of samples spiked with one *O*. *petrowi* egg. This qPCR assay is an effective assay to detect *O*. *petrowi* infections in wild birds. Our results suggest that fecal samples are the most appropriate sample for detecting infections; although, cloacal swabs can be useful for determining if *O*. *petrowi* is circulating in a population.

## Introduction

Parasites of the genus *Oxyspirura* (Spirurida: Thelaziidae) are heteroxenous nematodes that primarily use avian species as definitive hosts [1]. *Oxyspirura* spp. have been reported from more than 80 avian species worldwide [1]. *Oxyspirura petrowi* has recently received increased attention because of high prevalences detected in declining native Galliformes populations in North America [2,3]. *Oxyspirura petrowi* has a broad host range and has been reported infecting birds in the order Passeriformes and Galliformes [2,4–6]. Particularly, *O*. *petrowi* has been detected in high prevalences in northern bobwhites (*Colinus virginianus*) in western Texas [3], and lesser prairie chickens (*Tympanuchus pallidicinctus*) in southwestern Kansas [2,7]. Over the last century northern bobwhite and lesser prairie chicken populations have been going experiencing declines [8,9]. The cause of these decline has been largely attributed to habitat loss, habitat fragmentation, and climatic variables [10–12] with the role of disease only recently being investigated [3,7].

In wild birds infection with *O*. *petrowi* is known to cause lesions on the cornea and intraorbital glands [13]. These lesions could potentially affect vision of infected birds which may reduce their ability to escape predation and forage; however, this needs to be more thoroughly examined. Additionally, there is a lack of a validated diagnostic assays to detect *O*. *petrowi* infection in wild birds. Traditionally, fecal float techniques have been utilized [2]; however, problems arise with diagnostic sensitivity of these assays and proper identification of parasite eggs to species [14]. The objective of this study was to design a quantitative PCR (qPCR) assay to detect the presence of *O*. *petrowi* in samples collected from wild birds. We chose to developed a qPCR because it allows for quicker results and more accurate quantification over standard PCR protocols [15].

## Materials and Methods

### PCR Primer and Probe Design

To design primers specific to *O*. *petrowi*, we aligned the ITS2 locus of *O*. *petrowi* (GenBank accession nos. KF110800, KF110799, KF306222, and KT958839-KT958867). We selected the ITS region because it has been successfully used in previous qPCR studies and it has the most sequence information available [16, 17]. We then created a consensus sequence and used Primer Express v3.0.1 (Life Technologies Corp., Carlsbad, CA, USA) to design primers and hydrolysis probe (Table 1). We used the same method to design primers and a hydrolysis probe to the northern bobwhite NADH dehydrogenase 2 mitochondrial gene (ND2; GenBank accession no. KC556537; Table 1). For optimal amplification of our product, we only included primers with melt temperatures between 59–64°C and probes with melt temperatures ≥68°C [18].

**Table 1:** Primers and hydrolysis probes analyzed for quantitative PCR for *Oxyspirura petrowi* detection.

Primers	Sequence 5’-3’	Product
Oxy2448F	GTTTCCTCATGTGATTTCATTTTGT	
Oxy2597R	ATAAACGTTATTGTTGCCATATGCT	
Oxy_Probe_1	FAM- AAAGAAAGGTAATTCATCTGGT-MGB	149bp
ND2_70F	CAACCACTGAATCATAGCCTGAAC	
ND2_149R	GGTGGTGGGATTTTGAAATGAG	
Quail_ND2_Probe1	VIC- AGGAACCACAATCAC-MGB	79bp

### Standard Curve and Assay Optimization

To create a standard curve to determine qPCR efficiency we cloned a 244 base pair fragment using Qiagen PCR Cloning plus kit (Qiagen Inc., Germantown, MD). The DNA for cloning was extracted and sequenced in a previous study [16]. After cloning, we performed colony PCR on single white *Escherichia coli* colonies to ensure our locus was present. Then we purified the plasmids using QIAprep Spin Miniprep Kit (Qiagen Inc.) and sequenced confirmed to verify the presence of the ITS2 locus. We quantified plasmid DNA using QUBIT 3.0 Fluorometer (Thermo Fisher Scientific Inc., Waltham, MA, USA) and then calculated the plasmid copy number based on the size of the plasmid and insert. We then did serial dilutions from 5x10^5^-5x10^0^ for standard curves for each qPCR reaction.

All qPCR reactions were conducted in an Applied Biosystems StepOnePlus real time PCR machine (Thermo Fisher Scientific). In order to optimize the qPCR reactions, we ran each primer set using Powerup SYBR Green Master Mix (Thermo Fisher Scientific) with variable primer concentrations: 100 nM, 200 nM, 500 nM, 750 nM, and 1000 nM. We ran each primer concentration paired with itself (e.g. 100 nM forward and 100 nM reverse) and then paired with each other primer concentration (e.g. 200 nM forward and 1000 nM reverse). Each primer combination was run with standards ranging from 10^6^−10^1^ and standard curves were generated for comparison. We ran all standards in duplicate and the performance of primer pairs was evaluated using the StepOne Software v2.3 (Thermo Fisher Scientific), by calculating R^2^, slope, and estimating replication efficiency. Furthermore, we optimized the qPCR annealing temperature by running reactions with annealing temperatures varying from 58°C-60°C. We altered the reactions by 1°C at a time. The StepOnePlus system does not allow for multiple temperatures to be run in a single reaction; therefore, all reactions were run separate with the same standards and compared as describe for primer concentration fluctuation assays.

All SYBR green reactions were run with the following parameters: 95°C for 10 min; followed by 40 cycles of 95°C for 15s; and 60°C for 1 min. The cycle was followed by a melt curve analysis of 95°C then a gradient going from 60°C to 95°C in 0.3°C increments. Each reaction contained 10 μl Powerup SYBR Green Master Mix, variable amounts of 100 μm forward and reverse primer, 0.1 μl 10% BSA, variable amount of molecular grade water, and 2 μl of standard, making a final volume of 20 μl.

After we optimized the primer concentrations, we then tested the primer sets with hydrolysis probes to examine which primer and probe set worked most efficiently. Probe qPCR reactions were run with the following parameters: 50°C for 2 min; 95°C for 10 min; followed by 40 cycles of 95°C for 15 sec; and 60°C for 1 min. Each reaction contained 10 μl TaqMan Fast Advanced Master Mix (Thermo Fisher Scientific), 0.04 μl of 100 μm forward and reverse primer, 0.04 μl 100 μm probe, 0.1 μl 10% BSA, 7.82 μl molecular grade water, and 2 μl of each standard.

We used the northern bobwhite ND2 gene as an internal control for our extraction method and incorporated the primer and probe set in a duplex PCR. To determine if running a duplex PCR would impact our amplification, standards were run in triplicate as a duplex reaction and a single reaction (*O*. *petrowi* primers and probe only) in a single PCR reaction. We then compared slope and r^2^ values for the standards in both reactions. The duplex qPCR reaction was done with the same parameters as the hydrolysis probe PCR with the exception of adding 0.02 μl of each: ND2_70F; ND2_149R; Quail_ND2_probe1 and 7.76 μl of molecular grade water.

### Optimization of Fecal DNA Extraction

To optimize and determine detection limit of the extraction method, we collected *O*. *petrowi* eggs from fecal samples from birds experimentally infected with *O*. *petrowi* [16]. Briefly, after the fecal flotation technique, *O*. *petrowi* eggs were washed into a Petri dish. We then collected eggs using a pipette under a microscope at 100x magnification. We placed eggs into 2 ml microcentrifuge tubes with locking caps. We collected 15 replicates of 1 egg, 15 replicates of 2 eggs, and 15 replicates of 5 eggs. We then collected fecal swabs from uninfected pen-raised northern bobwhites to add to each microcentrifuge tube. Swab samples were used instead of fecal samples because swab samples were collected from wild northern bobwhites.

We tested our extraction method with three Qiagen extraction kits: Qiamp Fast Stool Minikit; Qiamp Stool Minikit; and DNeasy Plant Minikit. Prior to extraction, we added 2.4 mm metallic beads (6–8 beads, Omni International, Kennesaw, Georgia, USA) to each microcentrifuge tube with the swab. We then added 1.4 ml of ASL buffer (Qiamp Stool Minikit), 1.2 ml of Inhibit-X buffer (Qiamp Fast Stool Minikit), or 1 ml of sterile PBS (DNeasy Plant Minikit) and disrupted the sample in a Genie Disruptor (VWR, Houston, TX, USA) for 5 min. We then followed the manufacturer’s instructions, with the following exceptions: 1) we used the human DNA protocol with the Qiamp Fast Stool Minikit; and 2) transferred 500 μl of sample after Inhibit-X tablet spin and subsequently used 500 μl of AL buffer, and 500 μl of ethanol for the Qiamp Stool Mini Kit. All samples were eluted with 200 μl of AE buffer. In an attempt to increase our ability to extract DNA, we used our best performing method (Qiamp DNA Stool Minikit) and added a liquid nitrogen freeze thaw step prior to performing the extraction. This was done as described above with 10 replicates of 1 and 5 eggs. All animal use procedures were approved by the Texas Tech University Institutional Animal Care Use Committee (Protocol# 13066–08).

### Sample Testing

To analyze the ability to detect *O*. *petrowi* infections from birds, we collected paired fecal samples and cloacal swabs from wild caught northern bobwhites and from experimentally infected pen-raised birds [3,15]. We also conducted fecal floats on fecal samples from the experimentally infected birds using the Cornell-Wisconsin double centrifugation method. We extracted both fecal samples and cloacal swabs using the Qiamp DNA Stool Minikit using a liquid nitrogen freeze thaw. We used 180–220 mg of feces for fecal extractions and the mass of feces on swab samples was 48 mg (38-58mg 95% CI). The extractions were carried out as described above. We experimentally infected northern bobwhites with third stage *O*. *petrowi* larvae as described previously [15]. In total we infected 18 pen-raised northern bobwhites with third stage *O*. *petrowi* larvae and three birds were sham infected with Ringer’s solution. We also included a negative control (e.g. swab with no feces) every 12^th^ sample in every DNA extraction. All samples were run in duplicate and we considered a sample to be positive for *O*. *petrowi* if both replicates had a quantification cycle (Cq) <40. If only one replicate tested positive the sample was rerun. We also tested the qPCR assay against *Aulenocephalus pennula* a common nematode in wild quail from western Texas [18].

### Statistical Analyses

We compared agreement between the qPCR assays and fecal float assay using percent agreement and kappa statistics using agreement criteria based on Landis and Koch [19]. We used McNemar’s χ^2^ to compare the number of positive samples detected between qPCR fecal samples and qPCR cloacal swab samples. We compared Cq and egg per gram values from experimental infected birds using the non-parametric Spearman’s rank test. All statistical analyses were conducted using Rv3.2.4 (www.cran.r-project.org) with the fmsb package.

## Results

### qPCR Optimization

**Fig 1:**
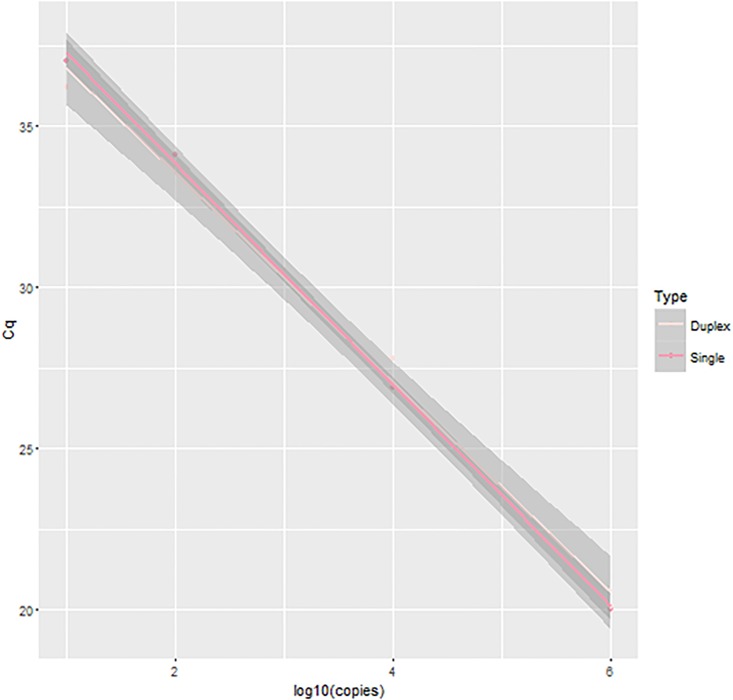
Standard curves from the duplex and single quantitative PCR reactions. Both curves were generated from standards (10^6^−10^1^) run in triplicate. For the duplex reaction r^2^ = 0.994 and the slope = -3.3 and for the single reaction the r^2^ = 0.998 and the slope = -3.4.

We did not detect any differences in the SYBR green PCR with primer concentrations ≥200 nM, however; standards run with 100 nM concentration forward or reverse primer had a lower efficiency. The optimal qPCR annealing temperature was 60°C. Therefore, we used a final concentration of 200 nM for the primers and an annealing temperature of 60°C. We then tested the *O*. *petrowi* qPCR assay with a hydrolysis probe as a single qPCR and as a duplex qPCR with the northern bobwhite ND2. We used six standard controls in triplicate and ran the single qPCR and duplex qPCR in the same reaction plate. We did not detect a decrease in assay efficiency with the duplex reaction compared to the single qPCR reaction (Fig 1). However, we did have increased variation in our 10 DNA copy standard (could not get all three samples within 1 Cq) and because of this variation we considered samples with less than 100 copies to be detectable but not quantifiable.

### Extraction Optimization

In order to determine the best extraction method from swab samples, we compared three different extraction protocols. The DNeasy Plant Mini kit extraction was able to detect positive samples in 2/5 one egg replicate, 2/5 two egg replicates, and 4/5 five egg replicates. The Qiamp Fast DNA Stool Minikit was able to detect positives in 1/5 one egg replicates, 3/5 two egg replicates, and 4/5 five egg replicates. The Qiamp DNA Stool Minikit detected positives in 4/5 one egg replicates, 4/5 two egg replicates, and 5/5 five egg replicates. In order to try and increase our ability to detect *O*. *petrowi* DNA from spiked swab samples, we used the Qiamp DNA Stool Minikit with a liquid nitrogen freeze thaw. This method detected positives in 10/10 one egg replicates and 10/10 five egg replicates. We did not observe any amplification from any of the negative controls.

### Sample Testing

**Fig 2:**
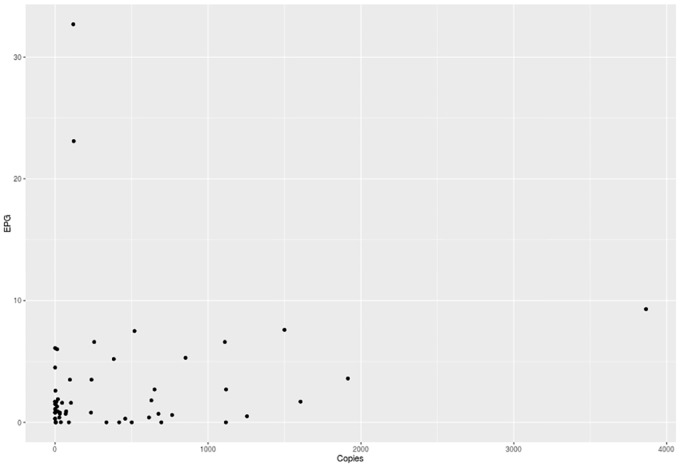
DNA copy number for the ITS2 region of *Oxyspirura petrowi* as determined by quantitative PCR on fecal samples and the corresponding egg per gram counts as determined by fecal flotation.

To determine the difference in extracting fecal samples from swab samples, we extracted paired fecal and cloacal swabs from wild birds (n = 29) and from experimentally infected birds (n = 90). In total, we detected *O*. *petrowi* DNA in 52.1% (62/119) fecal samples and 18.4% (22/119) swab samples with significantly more positive samples from fecal samples (Pearson’s χ^2^ = 30.4; p<0.01) and only slight agreement between sampling methods (Table 2). We also had corresponding fecal float data from the 90 experimentally infected bird samples. Overall, we detected *O*. *petrowi* eggs in 46.7% (42/90) of fecal samples by fecal flotation with an average detection of 3.5 eggs per gram (1.9–5.1 95%CI). There was substantial agreement between the fecal flotation results and qPCR results from fecal samples, but there was only slight agreement with the fecal flotation results and qPCR results from swabs (Table 2). We did detect a slight increasing trend in eggs per gram detected and ITS2 copy number from feces; however, this trend was not significant (Spearman's ρ = 0.13; p = 0.3; Fig 2). We did not amplify and DNA from *A*. *pennula* samples analyzed.

**Table 2:** Results from quantitative PCR (qPCR) on fecal samples and cloacal and fecal float results from experimentally infected and wild caught northern bobwhites (*Colinus virginianus*).

Sample Comparison	Number of samples	% Agreement	Kappa (95%CI)
qPCR Feces vs. Fecal Floats	90	84	0.69 (0.53–0.84)
qPCR Swabs vs. Fecal Floats	90	50	0.05 (-0.15–0.69)
qPCR Feces vs. qPCR swabs	119	58	0.18 (0.01–0.35)

## Discussion

Adequate diagnostic tests are a huge barrier to better understanding the epidemiology of wildlife diseases [20]. The qPCR we analyzed in this study is a sensitive assay with a low end detection of 1 egg in a sample. In addition, it provides a rapid and efficient method of antemortem testing of *O*. *petrowi* infection in wild birds. Prior to the development of this assay, the only antemortem testing for *O*. *petrowi* infection was fecal float analysis. Fecal float analyses can be difficult because it takes specialized training to properly identify parasite eggs and identification to species may never be accurate [14]. This assay eliminates the problematic identification and training associated with identifying parasite eggs.

One previous study has attempted to design a qPCR for the detection of *O*. *petrowi* and showed success in amplifying *O*. *petrowi* DNA from fecal samples [21]. We analyzed these primers for our study, but did not use them for several reasons. The first is the annealing temperature for the qPCR reaction was only 56°C, which is low for hydrolyses probe qPCR [22]. Second the primer QEW_2417F covers a region in the ITS2 locus that contains a polymorphic region. Both of these factors would likely reduce the amplification efficiency of this assay.

The low detection levels from cloacal swabs compared to fecal samples is probably related to the amount of fecal material contained on the swab sample. In this study the mass of feces on a swab sample was approximately 25% of the material used for a fecal extraction. This likely reduces the number of eggs collected in a sample and therefore reduces our ability to detect infection. A previous study examining the amount of target DNA recovered from fecal samples and fecal swabs showed DNA extracted from fecal samples contained ~14x more target DNA than that from fecal swabs [23].

Our comparison between the egg per gram levels (fecal flotation) and *O*. *petrowi* ITS2 copy number only showed a weak positive correlation. This is probably due to a combination of the inability of the assay to accurately quantify samples with less 100 ITS2 copies and the low egg per gram levels we detected. However, the strong agreement between the results of qPCR on fecal samples and the fecal flotation methods suggests this qPCR assay is effective at detecting even low level *O*. *petrowi* infections from fecal samples. Additionally, we were able to amplify *O*. *petrowi* DNA in all of our experimental extractions where as little as one *O*. *petrowi* egg was included in the extraction.

Besides the qPCR assay, we also validated an extraction method for *O*. *petrowi* from fecal swabs. All three kits we used were able to detect one egg in a spiked cloacal swab sample. However, only the Qiamp DNA Stool Minikit was able to amplify 4/5 of the one egg replicates without a liquid nitrogen freeze thaw and 10/10 one egg replicates with the liquid nitrogen freeze thaw. This is in contrast to previous studies that showed the DNeasy Plant Minikit to be the most effective method for extraction of DNA from nematode eggs in fecal samples [24, 25]. These results further highlight the need to validate nucleic acid extraction methods along with validating the qPCR reaction in order to optimize the assay.

This study shows an effective method for detecting *O*. *petrowi* infections from fecal and swab samples in wild birds. It is clear that fecal samples are the best method for detection *O*. *petrowi* infections; however, swab samples are easy to collect and require less time handling birds to reduce stress. For these reasons, swab samples can be preferably used to examine whether *O*. *petrowi* is currently circulating at a population level. Due to this qPCR assay being able to detect as few as one egg in a sample, it can be used as an effective tool to detect *O*. *petrowi* infections in populations of wild birds.
